# Oral Delivery of *Lactococcus lactis* Expressing Full-Length S Protein via Alginate–Chitosan Capsules Induces Immune Protection Against PEDV Infection in Mice

**DOI:** 10.3390/vaccines13040421

**Published:** 2025-04-17

**Authors:** Miaoyan Yang, Denglong Xie, Wei Ji, Shu Jeffrey Zhu, Yongqi Zhou

**Affiliations:** 1 Department of Veterinary Medicine, College of Animal Sciences, Zhejiang University, Hangzhou 310058, China; miaoyanyang@outlook.com; 2 Zhejiang Hisun Animal Healthcare Products Co., Ltd., Hangzhou 311400, China; 3 Yunnan Biopharmaceutical Co., Ltd., Kunming 650599, China

**Keywords:** adjuvant, immune response, *Lactococcus lactis*, oral vaccine, PEDV

## Abstract

**Background/Objectives**: Porcine epidemic diarrhea (PED) is a highly contagious enteric infectious disease that causes severe morbidity and mortality in piglets, posing significant economic losses to the swine industry worldwide. Oral vaccines based on *Lactococcus lactis* offer a promising approach due to their safety and genetic manipulability. This study aims to develop and evaluate an oral *L. lactis*-based vaccine expressing the full-length PEDV S protein. **Methods**: A recombinant *L. lactis* strain expressing the PEDV S protein was constructed and encapsulated in alginate–chitosan microcapsules. Vaccine stability was tested in simulated digestive fluids, and mice were orally immunized. Immune responses were evaluated by measuring specific antibodies, cytokines, and lymphocyte proliferation. **Results**: The recombinant *L. lactis* NZ3900/pNZ8149-S strain successfully expressed the full-length PEDV S protein and maintained stable plasmid inheritance. Oral immunization in mice induced detectable PEDV-specific immune responses. Both encapsulated and non-encapsulated vaccines stimulated the production of IgG and sIgA antibodies, as well as cytokines associated with Th1 and Th2 responses. Notably, encapsulation with alginate–chitosan significantly enhanced bacterial survival in digestive conditions and further amplified immune responses, including higher antibody titers, elevated levels of IFN-γ, IL-4, and IL-10, and greater lymphocyte proliferation, indicating improved immune memory. **Conclusions**: The oral *L. lactis* NZ3900/pNZ8149-S vaccine expressing the PEDV S protein effectively induced systemic and mucosal immunity in mice. Encapsulation with alginate–chitosan further enhanced its immunogenicity and stability in gastrointestinal conditions. These results suggest that both the engineered *L. lactis* strain and the encapsulation strategy contribute to the development of a promising oral vaccine platform for controlling PEDV in swine populations.

## 1. Introduction

Porcine epidemic diarrhea virus (PEDV) is the primary pathogen responsible for porcine epidemic diarrhea (PED), a severe gastrointestinal disease marked by diarrhea, vomiting, and dehydration, with mortality rates over 95% in neonatal piglets [1,2]. First detected in Europe in the early 1970s [3], PEDV has since become a global concern, with highly pathogenic PEDV variants emerging since 2010, leading to significant economic losses in the swine industry [4]. Despite recent advancements in prevention, PEDV continues to pose a major challenge [5,6,7]. The spike (S) protein of PEDV plays a crucial role in the virus’s ability to infect host cells, making it a key target for vaccine development. The S protein is composed of two major domains, S1 and S2 [8]. The S1 domain mediates viral entry by binding to host cell receptors and inducing neutralizing antibodies, while the S2 domain is responsible for facilitating membrane fusion [9,10]. Within the S1 region, the C-terminal domain (COE) and the N-terminal domain (NTD) are critical for inducing neutralizing antibodies and may serve as key targets for vaccine development [11,12]. Consequently, the full-length S protein, along with its subdomains (S1 and COE), have been extensively explored in the design of PEDV vaccines. Furthermore, studies comparing vaccines encoding the full-length S protein versus its subunits have shown that the full-length S protein induces significantly higher levels of neutralizing antibodies, suggesting its superiority as a vaccine target [13,14]. The continuous evolution of PEDV has further complicated vaccine development. Variants of PEDV have shown increased pathogenicity, enhanced transmissibility, and altered antigenicity, making existing vaccines less effective against emerging strains [2,4]. Vaccine strategies must, therefore, not only focus on inducing robust immunity, but also account for the genetic diversity of PEDV. The inclusion of conserved antigenic regions in vaccine design has been proposed as a potential approach to address this challenge, as these regions are less likely to undergo significant mutations [15].

While traditional non-oral vaccines induce systemic immunity, they primarily stimulate IgG production in the bloodstream, an approach that is inadequate for neutralizing PEDV in the intestines [3,16]. This stresses the need for oral vaccines, which can induce both systemic IgG and mucosal immunity through the secretion of secretory IgA (SIgA) in the intestinal mucosa, offering enhanced protection against infection [3,17,18]. The induction of mucosal immunity is particularly important for combating PEDV, as the virus primarily infects the intestinal epithelial cells. SIgA, the predominant antibody in mucosal secretions, plays a crucial role in neutralizing pathogens at mucosal surfaces and preventing their adhesion and invasion [3]. Despite their potential advantages, oral vaccines face significant challenges in delivery and efficacy. The harsh conditions of the gastrointestinal tract, including the low pH, digestive enzymes, and bile salts, can degrade the vaccine components, reducing their stability and immunogenicity [19,20]. Additionally, achieving a sufficient uptake of antigens by mucosal immune cells is a critical factor for the success of oral vaccines. Strategies to enhance the stability and delivery of oral vaccines are, therefore, essential for their effective application. Live vector vaccines have emerged as a promising approach to address these challenges. These vaccines utilize live microorganisms, such as bacteria or viruses, to deliver antigens to the host immune system. Among these, recombinant *Lactococcus lactis* has garnered significant attention due to its safety profile, ease of genetic manipulation, and ability to survive transiently in the gastrointestinal tract [21,22]. *L. lactis* has been widely used as a live delivery vector for various antigens, demonstrating its potential to induce robust mucosal and systemic immune responses [23,24,25].

The stability of live probiotic vaccines is a critical factor for ensuring effective antigen delivery and immune response [26]. To improve antigen delivery to mucosal surfaces, encapsulating agents like sodium alginate and chitosan are often employed [27,28,29,30]. Sodium alginate, a natural material, can form hydrogels in the presence of divalent cations like Ca^2+^ [31,32]. Chitosan, a natural polysaccharide, offers excellent biocompatibility, biodegradability, and adhesion, along with a protective barrier against environmental stresses [33]. Chitosan has been widely studied for its potential to induce strong immune responses, particularly in mucosal immunity. It has also been successfully tested in various vaccine formulations [34]. Combining sodium alginate and chitosan into capsules has been shown to provide protection and stability for probiotics during digestion. Encapsulation technology has advanced significantly in recent years, offering innovative solutions for improving the delivery and efficacy of oral vaccines. Alginate–chitosan capsules have been extensively studied for their ability to protect antigens from the harsh gastrointestinal environment, ensuring their controlled release at the desired site of action [35]. The incorporation of divalent cations, such as calcium ions, further enhances the structural integrity of the capsules, preventing premature release of the encapsulated antigens [36]. Recent studies have demonstrated the efficacy of alginate–chitosan capsules in improving the stability and immunogenicity of oral vaccines. The use of chitosan as an adjuvant has also been reported to enhance the uptake of antigens by dendritic cells and other antigen-presenting cells, further boosting the immunogenicity of the vaccine [37,38]. Compared to other polymer-based vaccine delivery systems, such as poly (lactic-co-glycolic acid) (PLGA) or liposome-based carriers, alginate–chitosan capsules offer several advantages, including cost-effectiveness, non-toxicity, and efficient oral delivery [39]. These capsules also provide a controlled release mechanism that ensures the gradual release of antigens in the intestine, where mucosal immune responses are crucial [40,41]. In addition, the combination of sodium alginate and chitosan overcomes the limitations of each individual polymer. For instance, while sodium alginate protects antigens from gastric degradation [42], chitosan enhances antigen uptake and facilitates stronger mucosal immunity [43,44].

In this study, alginate–chitosan capsules were developed to encapsulate recombinant *L. lactis* expressing PEDV antigens. The stability of the encapsulated vaccine was evaluated under simulated digestive conditions, and its immunogenic efficacy in mice was assessed. This study offers a promising strategy for PEDV prevention and could advance the application of encapsulated recombinant *L. lactis* vaccines in the swine industry. By leveraging the unique properties of alginate–chitosan capsules, this approach addresses the challenges of oral vaccine delivery, providing a robust platform for inducing mucosal and systemic immunity against PEDV. Moreover, the implications of this research extend beyond PEDV. The development of effective oral vaccine delivery systems has broad applications for combating other gastrointestinal pathogens in both humans and animals. The use of biocompatible and biodegradable materials, such as alginate and chitosan, emphasizes the potential for safe and scalable vaccine production. Future studies could explore the incorporation of additional adjuvants or modifications to the capsule composition to further enhance vaccine efficacy.

## 2. Materials and Methods

### 2.1. Construction of Recombinant L. lactis Strains

**Figure 1:**
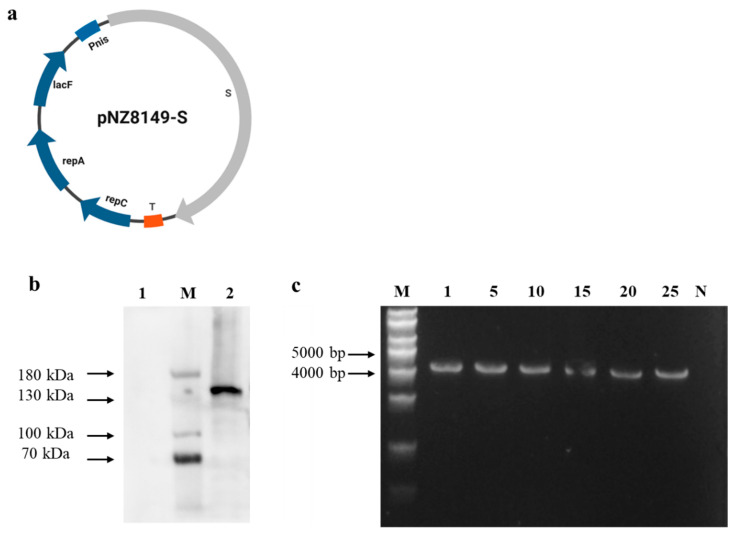
Construction, expression, and stability of the pNZ8149-S plasmid in *L. lactis* NZ3900. (**a**) The construction of the pNZ8149-S plasmid is as described in the article. (**b**) The expression of the S protein in *L. lactis* NZ3900 was analyzed by western blotting. Lane 1 shows NZ3900 with the plasmid pNZ8149 with nisin induction; M represents the protein marker. Lane 2 shows NZ3900 with the plasmid pNZ8149-S induced with nisin. (**c**) PCR detection of the S gene in various generations of recombinant *L. lactis* NZ3900/pNZ8149-S.M: DNA marker; lanes 1-25: PCR products from different generations of recombinant *L. lactis* NZ3900/ pNZ8149-S; N: PCR product of wild-type *L. lactis* NZ3900/pNZ8149 (negative control).

*L. lactis* NZ3900 and pNZ8149 were obtained from Honorgene (Changsha, China). The S gene was synthesized by Sangon Biotech (Shanghai, China) based on a variant PEDV strain (*PEDV/CHN/SHANGHAI/2012* (*SH 2012*); GenBank: MG837011). Initially, the sequence of the PEDV S gene was amplified using the primer pair PEDVS-F: 5′-aaattataaggaggcactcaATGAAATCTTTAACATATTTTTGGTTATTTT-3′ and PEDVS-R: 5′-taattttggttcaaagaaagTTAATGGTGATGGTGATGGTGTTGAACATGAACTTTTTCAAAAACTTC-3′. Then, the expression vector pNZ8149 was digested using the primers pNZ8149_F: 5′-ctttctttgaaccaaaattagaaaacc-3′ and pNZ8149_R: 5′-tgagtgcctccttataatttattttgt-3′, purified with the Universal DNA Purification Kit (TIANGEN, Beijing, China) and ligated with the S gene to generate the recombinant plasmid pNZ8149-S using the ClonExpress Ultra One Step Cloning Kit (Vazyme Biotech, Nanjing, China) (Figure 1a).

The recombinant plasmid (pNZ8149-S) was transformed into *L. lactis* NZ3900 using electroporation based on previously established methods [45]. Electroporation was performed using a Bio-Rad Gene Pulser (Bio-Rad, Hercules, CA, USA). Positive clones were identified through PCR amplification (PEDVS-F/R) and confirmed by sequencing. The resulting positive recombinant *L. lactis* bacterium was designated as *L. lactis* NZ3900/pNZ8149-S, while the control strain carrying the empty plasmid was named pNZ8149/NZ3900. To assess the stability of the recombinant plasmids in *L. lactis* NZ3900, the transformed bacteria were passaged twenty times in GM17 broth without antibiotics. Every fifth generation was monitored for stability using PCR with primers PEDVS_F and PEDVS_R.

### 2.2. Preparation of Alginate–Chitosan Encapsulated L. lactis

**Figure 2:**
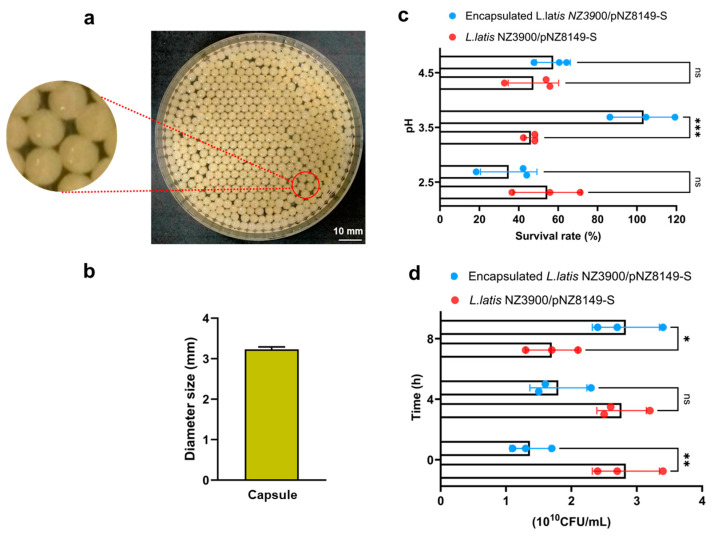
Characterization of encapsulated *L. lactis* NZ3900/pNZ8149-S capsules and evaluation of their resistance to various digestive conditions. (**a**) Encapsulated *L. lactis* NZ3900/pNZ8149-S: the image was captured using a camera. (**b**) Measurement of encapsulated *L. lactis* NZ3900/pNZ8149-S diameter: the diameter of the capsules was measured by randomly selecting three fields. (**c**) Stability of encapsulated *L. lactis* NZ3900/pNZ8149-S in simulated gastric fluid at pH levels 2.5, 3.5, and 4.5, incubated at 37 °C for 3 h. (**d**) Stability of encapsulated *L. lactis* NZ3900/pNZ8149-S in simulated intestinal fluid at time points 0 h, 4 h, and 8 h, incubated at 37 °C. Data are presented as the mean ± SD (*n* = 3). * indicates *p* < 0.05, ** indicates *p* < 0.01, *** indicates *p* < 0.001, and “ns” indicates no significance.

The study used chitosan (degree of deacetylation > 90%) from Macklin (Shanghai, China) and sodium alginate from Sinopharm Chemical Reagent Co., Ltd. (Shanghai, China) at a 1% (wt/vol) concentration each to prepare alginate–chitosan-encapsulated *L. lactis* following the method described by Rocha et al. [46], with slight modifications. The recombinant *Lactococcus* strain *L. lactis* NZ3900/pNZ8149-S was cultured overnight in M17 broth at 30 °C under anaerobic conditions for 16 h, and then harvested by centrifugation (4000 rpm, 10 min, 4 °C). The cell pellet was then gently mixed with an alginate and chitosan mixture to a final concentration of 10^11^ CFU/mL. Next, the resulting bacterial alginate–chitosan solution was then dripped into a calcium chloride solution (8%, wt/vol) using a syringe, forming spherical capsules (Figure 2a). To evaluate tolerance to the intestinal fluid environment, *L. lactis* NZ3900/pNZ8149-S was incubated in 5 mL of simulated intestinal fluid (SIF) containing trypsin at 37 °C for 8 h, with measurements taken at 4 h intervals. Additionally, the tolerance of *L. lactis* NZ3900/pNZ8149-S to gastric juice was assessed in vitro. *L. lactis* NZ3900/pNZ8149-S was incubated in 5 mL of simulated gastric juice (SGF) containing pepsin, adjusted to pH levels of 2.5, 3.5, or 4.5, and maintained at 37 °C for 3 h. For comparison, an unencapsulated live recombinant strain, *L. lactis* NZ3900/pNZ8149-S, served as the control. Each experiment was repeated three times, and viable bacterial counts were determined using the plate count method.

### 2.3. Western Blotting

To assess the expression of the S protein, the bacteria were then washed with cold PBS and lysed using a SCIENTZ-IID sonicator (Scientz Biotechnology, Ningbo, China). Equal amounts of protein were separated by SDS-PAGE and transferred to a Sequi-Blot polyvinylidene difluoride (PVDF) membrane (Bio-Rad). The membranes were blocked with 5% skimmed milk for 1 h at room temperature and subsequently incubated with an anti-PEDV-S monoclonal antibody (Qianxun Biotech Company Co., Ltd., Guangzhou, China). Following three washes with TBST, the membranes were incubated with an HRP-conjugated goat anti-mouse secondary antibody (Beyotime, Beijing, China) for 1 h at room temperature. After additional washing, protein bands were visualized using immobilon western chemiluminescent HRP substrate (Merck Millipore, Billerica, MA, USA) for 5 min.

### 2.4. Immunization Schedule and Samples Collection

**Figure 3:**
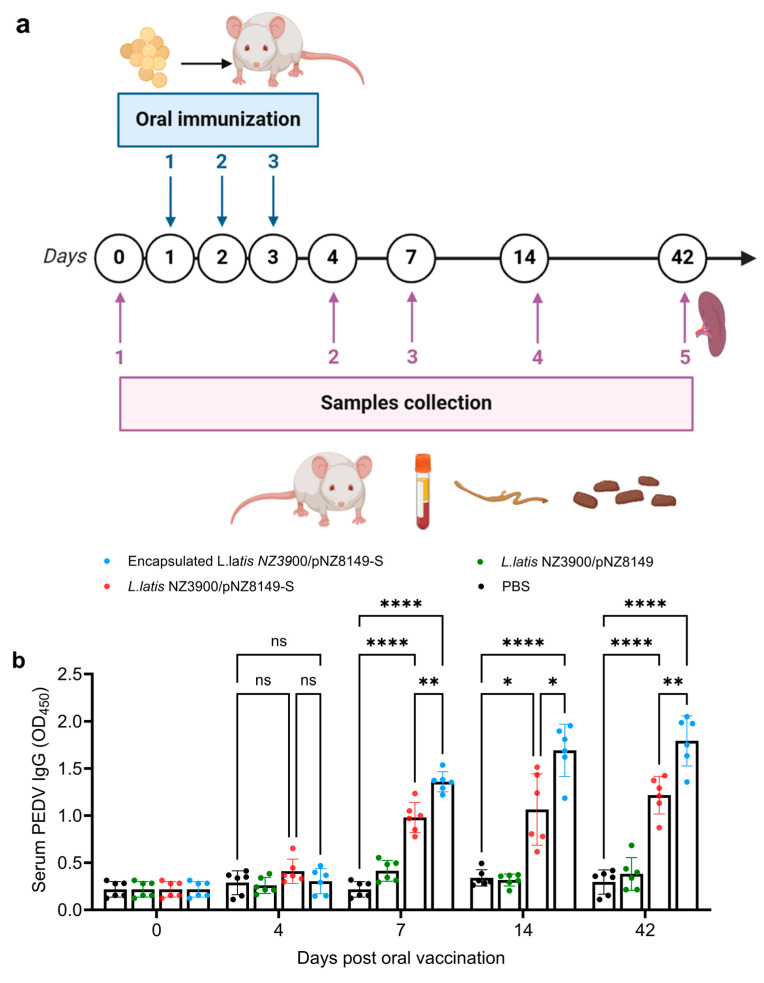

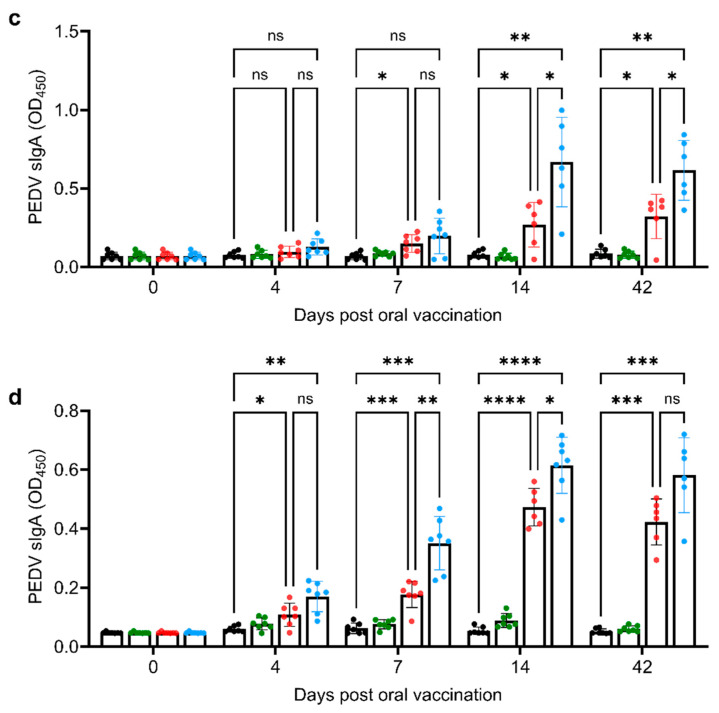
Antibody response after oral vaccine immunization in mice. (**a**) Experimental strategy for oral vaccination and monitoring of immune responses in mice. Samples were collected on days 0, 4, 7, 14, and 42 for analysis. (**b**) Levels of anti-PEDV IgG antibodies in groups of mice immunized with PBS, *L. lactis* NZ3900/pNZ8149, oral vaccine *L. lactis* NZ3900/pNZ8149-S, and encapsulated *L. lactis* NZ3900/pNZ8149-S, monitored at 0, 3, 7, 14, and 42 days post-immunization (dpi). The levels of anti-PEDV SIgA antibodies in the same groups were assessed at 0, 3, 7, 14, and 42 dpi. Intestinal mucus (**c**) and fecal (**d**) data are presented as the mean ± SD (*n* = 6). * indicates *p* < 0.05, ** indicates *p* < 0.01, *** indicates *p* < 0.001, **** indicates *p* < 0.0001, and “ns” indicates no significance.

The immunization schedule is shown in Figure 3a. One hundred two six-week-old female BALB/c mice (SPF, Beijing Biotechnology Co., Ltd., Beijing, China) were randomly divided into four groups, with 24 mice in each group, and the remaining six mice were sampled as Day 0. The first group was orally immunized with capsules containing alginate–chitosan-encapsulated *L. lactis* NZ3900/pNZ8149-S at a dose of 2 × 10^10^ CFU. The second group received an oral suspension of 2 × 10^10^ CFU recombinant *L. lactis* NZ3900/pNZ8149-S in 200 μL. The third group was orally administered a suspension of 2 × 10^10^ CFU recombinant *L. lactis* NZ3900/pNZ8149 in 200 μL. The final group received 200 μL of sterile phosphate-buffered saline (PBS) orally. All mice were boosted on days 2 and 3 after the initial immunization using the same methods. Blood samples were collected via tail bleeding on days 4, 7, 14, and 42 post-immunization and stored at −20 °C until analysis. Fecal and intestinal mucus samples were collected at the corresponding time points and processed based on previously described methods, with minor modifications [47].

### 2.5. Antibody and Cytokine Assay by Indirect Enzyme-Linked Immunosorbent Assay (ELISA)

Serum levels of antigen-specific IgG and sIgA were obtained from previously published protocols [48]. The levels of IgG in serum and sIgA in the contents of the small intestine and feces were determined using mouse-derived PEDV-IgG and PEDV-sIgA ELISA kits, in accordance with the manufacturer’s instructions (Goybio, Shanghai, China). The levels of IL-4, IL-10, and IFN-γ in these samples were quantified using commercial ELISA kits (Sangon, Shanghai, China), following the manufacturer’s guidelines. Cytokine concentrations were determined by referencing standard curves generated from the provided reagents, and the optical density (OD) at 450 nm was measured using a TECAN Infinite 200 PRO microplate reader (TECAN, Männedorf, Switzerland) to calculate cytokine levels.

### 2.6. Lymphocyte Proliferation Assay

As shown in Figure 3a, at 42 days post-vaccination, mice were harvested for the proliferation assay. In brief, 100 µL of the cell suspension (containing 5 × 10⁶ cells/mL) was added to each well of a 96-well plate, followed by stimulation with 5 µg/mL of recombinant PEDV-S protein. Six replicate wells were used for each sample. The plate was then incubated at 37 °C in a 5% CO₂ atmosphere for approximately 72 h. To assess cell proliferation, 10 µL of MTT solution (Beyotime, Beijing, China) was added to each well, followed by incubation for an additional 2 h at 37 °C. Absorbance at 570 nm (OD_570_) was measured, and the relative stimulation index (SI) was calculated by dividing the average OD value of antigen-stimulated wells by that of unstimulated controls.

### 2.7. Statistical Analysis

Statistical analysis and figure creation were performed using GraphPad Prism 10.4 software. Data are presented as the mean ± SD. Group differences were assessed for statistical significance using a two-way ANOVA followed by Tukey’s multiple comparisons. In the figures, significant differences are indicated by asterisks, with * indicating *p* < 0.05, ** indicating *p* < 0.01, *** indicating *p* < 0.001, **** indicating *p* < 0.0001, and “ns” indicating no significance, i.e., *p* > 0.05. The sample size (*n*) is provided in the respective figure legends.

## 3. Results

### 3.1. Identification of Recombinant L. lactis NZ3900

To develop the oral vaccine, full-length S protein was cloned into expression plasmid pNZ8149 (Figure 1a), the constructed plasmid was introduced into *L. lactis* NZ3900, generating *L. lactis* NZ3900/pNZ8149-S. The strain cultures were collected and analyzed using SDS-PAGE followed by immunoblotting. As anticipated, the recombinant S protein (~153 kDa) was detected in the *L. lactis* NZ3900 strains carrying the recombinant plasmid after induction with nisin (Figure 1b). In contrast, no protein bands were observed in the nisin-uninduced *L. lactis* NZ3900 strains or in the nisin-induced *L. lactis* NZ3900 strains containing the plasmid pNZ8149 (negative control lanes). To assess the stability of the pNZ8149-S plasmid in the recombinant *L. lactis* NZ3900, the positive colonies were cultured over 25 generations, followed by PCR analysis, confirming the presence of the expected genes (Figure 1c). Agarose gel electrophoresis revealed 4219 bp bands, indicating that the recombinant plasmid was stably inherited in *L. lactis* NZ3900 without the use of antibiotics in the medium. Furthermore, stable expression of the S protein was observed across various generations of recombinant *L. lactis* NZ3900/pNZ8149-S (Figure S1).

### 3.2. Characterization and Performance of Capsule Vaccine in Digestive Environments

Alginate–chitosan has proven to render probiotics stable, effectively supporting probiotic colonization in the gastrointestinal tract [49,50]. Based on this, the *L. lactis* NZ3900/pNZ8149-S vaccine was encapsulated using alginate–chitosan, producing *L. lactis* NZ3900/pNZ8149-S capsules designed for oral administration. An injector was employed to regulate the average particle size. The liquid from the injector dripped into the calcium chloride solution, forming spherical capsules that remained suspended in the solution. The resulting capsules displayed a granular morphology with uniform particle distribution and a consistent, homogenous structure. The microsphere membranes appeared smooth, intact, and well-formed (Figure 2a). The particles, composed of alginate–chitosan, were uniform in size, with an average diameter of approximately 3 mm (Figure 2b). Furthermore, the encapsulated live oral vaccine *L. lactis* NZ3900/pNZ8149-S was evaluated for its resilience against various digestive stresses, including exposure to SIF and SGF. The findings demonstrated that the alginate–chitosan capsules provided effective protection for the live probiotic vaccine under these harsh conditions. Specifically, for the encapsulated *L. lactis* NZ3900/pNZ8149-S group, the viability release rate was around 50% after 3 h in SGF at pH values of 2.5−4.5 (Figure 2c). In contrast, in SIF, the viability increased from 1.4 log CFU/mL to 2.8 log CFU/mL between 2 and 8 h (Figure 2d), indicating that the alginate–chitosan capsules effectively shielded the probiotic vaccine from digestive stress. Capsules protect lactic acid bacteria from the damaging effects of gastric acid, allowing for slow release in the intestinal fluid.

### 3.3. Alginate–Chitosan L. lactis NZ3900/pNZ8149-S Vaccine Induced Higher PEDV-Specific IgG and sIgA Antibodies in Mice

SIgA serves as the first line of defense in the immune response by blocking pathogens from entering through mucosal surfaces [51]. Therefore, we evaluated the systemic and mucosal antibody responses elicited by the candidate vaccines. IgG levels in serum and sIgA levels in fecal and intestinal mucus were measured following vaccination (Figure 3a). Starting on day 7, significantly higher specific anti-PEDV IgG antibody levels (*p* < 0.01) were detected in mice vaccinated with *L. lactis* NZ3900/pNZ8149-S (Figure 3b). Furthermore, mice immunized with alginate–chitosan-encapsulated *L. lactis* NZ3900/pNZ8149-S exhibited significantly higher systemic and mucosal antibody responses compared to those receiving *L. lactis* NZ3900/pNZ8149-S alone. Notably, after continued immunization, mucosal sIgA levels increased markedly in mice receiving either *L. lactis* NZ3900/pNZ8149-S or alginate–chitosan-encapsulated *L. lactis* NZ3900/pNZ8149-S vaccines compared to both the *L. lactis* NZ3900/pNZ8149 control group and the PBS mock group (Figure 3c,d). Moreover, PEDV-specific sIgA levels in the alginate–chitosan-encapsulated *L. lactis* NZ3900/pNZ8149-S group were significantly higher than those in the *L. lactis* NZ3900/pNZ8149-S group 14 days post-immunization, suggesting that the *L. lactis* NZ3900/pNZ8149-S vaccine effectively induces mucosal immunity in mice. These findings indicate that oral recombinant *L. lactis* NZ3900/pNZ8149-S vaccines promote both humoral and mucosal immunity, with the alginate–chitosan formulation inducing higher antibody levels.

### 3.4. Alginate–Chitosan L. lactis NZ3900/pNZ8149-S Vaccine Induced Significantly Increased IFN-r, IL-4, and IL-10 Cytokine in Vaccinated Mice

**Figure 4:**
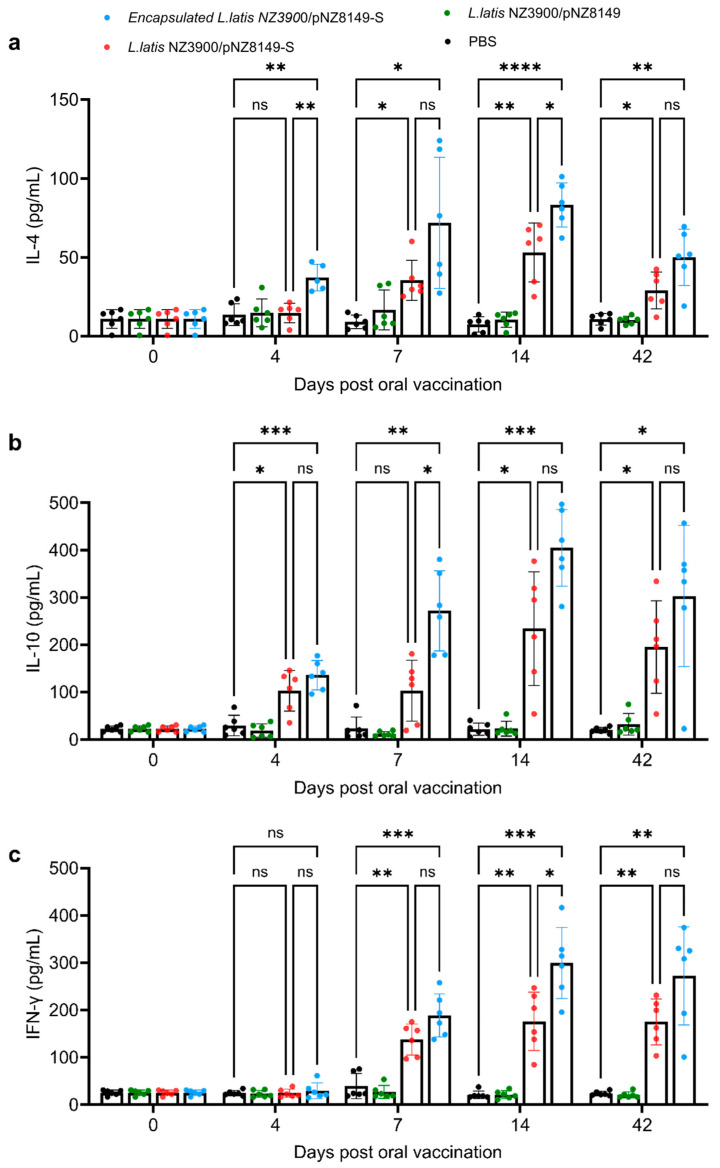
Cytokine levels were measured in the serum of immunized mice in the groups of mice immunized with PBS, *L. lactis* NZ3900/pNZ8149, the oral vaccine *L. lactis* NZ3900/pNZ8149-S, and encapsulated *L. lactis* NZ3900/pNZ8149-S. IL-4 (**a**), IL-10 (**b**), and IFN-γ (**c**) serum samples were collected on days 0, 4, 7, 14, and 42, either before or after immunization. Data are presented as the mean ± SD (*n* = 6). * indicates *p* < 0.05, ** indicates *p* < 0.01, *** indicates *p* < 0.001, **** indicates *p* < 0.0001, and “ns” indicates no significance.

To further characterize the cellular immune responses induced by the oral vaccines, cytokine production was assessed following immunization. We characterized the levels of three cytokines (IFN-γ, IL-4, and IL-10) in the serum of mice. Starting from day 4, both IL-4 and IL-10 levels were significantly higher in the orally vaccinated groups compared to both the *L. lactis* NZ3900/pNZ8149 control group and the PBS mock group (Figure 4a,b). Starting from day 7, IFN-γ levels also exceeded those in both the *L. lactis* NZ3900/pNZ8149 control group and the PBS mock group (Figure 4c). All cytokines peaked at day 14 and showed a slight decline by day 42 (Figure 4). These data suggest that both *L. lactis* NZ3900/pNZ8149-S capsules and *L. lactis* NZ3900/pNZ8149-S vaccines are effective in inducing cytokine responses in mice. Moreover, the cytokine analysis of blood samples collected on days 4, 7, 14, and 42 post-immunization revealed distinct immune responses induced by the recombinant *L. lactis* NZ3900/pNZ8149-S vaccines and the encapsulated version. The recombinant *L. lactis* NZ3900/pNZ8149-S vaccines stimulated an early increase in the Th2 cytokines IL-4 and IL-10, both of which peaked on day 14 and gradually declined by day 42, suggesting a transient humoral response. In contrast, the encapsulated vaccine induced a stronger and more sustained IFN-γ response, indicative of a more prominent Th1-type cellular immune response, with peak levels observed on day 14 and remaining elevated through day 42. The enhanced IFN-γ production in the encapsulated vaccine group underscores the adjuvant potential of the alginate–chitosan capsule formulation in promoting cellular immunity.

### 3.5. The Lymphocytes Proliferation

Lymphocyte proliferation plays a critical role in the immune response against PEDV, as it enhances the ability of the immune system to recognize and combat the virus [52,53]. The proliferation of T lymphocytes (both CD4+ and CD8+ T cells) and B lymphocytes is central to generating an adaptive immune response [54,55]. To further assess the impact of capsules on cell-mediated immunity, splenocytes were isolated and re-stimulated in vitro to evaluate cellular immune responses at 42 days post-immunization to assess immune memory in relation to PEDV. As shown in Table 1, the stimulation indices of the encapsulated *L. lactis* NZ3900/pNZ8149-S and *L. lactis* NZ3900/pNZ8149-S immunization groups were significantly higher compared to both the *L. lactis* NZ3900/pNZ8149 control group and the PBS mock group (*p* < 0.05), indicating that both the oral S vaccine and the chitosan-based S vaccines elicited significantly higher lymphocyte proliferation. Among these, the proliferative rate in the *L. lactis* NZ3900/pNZ8149-S capsules was notably greater than that observed in the *L. lactis* NZ3900/pNZ8149-S group. These results suggest that the capsule vaccine immunization strategy induces a stronger lymphocyte proliferation response in mice, contributing to immune memory for the immune response against PEDV.

## 4. Discussion

PED is a highly contagious disease characterized by its preference for intestinal tissues [56]. *Lactococcus*, as a naturally occurring intestinal bacterium that secretes proteins, can be combined with immune adjuvants and target antigens to sustain immune stimulation at the mucosal surface, offering a promising strategy for achieving mucosal immunity against PEDV. However, some difficulties still remain with respect to developing an oral vaccine against PEDV. For the development of oral vaccines, it is crucial to identify the optimal protective antigen gene, which serves as the foundation for creating genetically engineered vaccines. It has been established that the S protein of PEDV is a key target for neutralizing antibodies, as it plays a crucial role in mediating the interaction between the virus and host cell receptors, thereby facilitating viral entry into cells [57]. Moreover, the use of adjuvants significantly enhances the efficacy of subunit protein vaccines by boosting the immune response [58]. Alginate–chitosan capsules are increasingly recognized for their low cytotoxicity and excellent biocompatibility, making them an ideal carrier for the controlled release of vaccine antigens [59]. Chitosan, in particular, has demonstrated the ability to stimulate robust immune responses, and its potential has been widely explored in various vaccine formulations [60]. Here, we developed a capsule-based *L. lactis* oral vaccine by constructing a *Lactococcus* expression plasmid for PEDV, which was created by inserting the PEDV S protein gene (Figure 1). The recombinant *Lactococcus* strain expressing the PEDV S protein was then mixed with sodium alginate and chitosan adjuvants to prepare the *L. lactis* NZ3900/pNZ8149-S capsule vaccine (Figure 2).

In previous studies, oral lactis vaccines have been typically administered to mice or pigs at two-week intervals, with antibodies against PEDV observed three weeks post-immunization [48,61,62]. However, this immunization schedule poses challenges for combating PEDV, particularly in neonatal pigs. PEDV is highly fatal in newborn piglets, often causing death within a week of infection [2]. To address this urgent need for faster immunity, our study explored a more aggressive immunization protocol. We administered the oral lactis vaccine expressing the full-length S protein of PEDV over three consecutive days. This rapid, consecutive dosing schedule was designed to accelerate the immune response compared to the standard two-week interval. Our findings showed that antibodies against PEDV were observed after 7 days of the first vaccination (Figure 3), demonstrating that the intensified vaccination schedule can provoke a quicker immune response. This accelerated immune response is particularly crucial for applications in newborn piglets, where the high mortality rate from PEDV makes rapid immunity essential. By generating protective antibodies within a week, this approach has the potential to improve survival rates in piglets. The lactis vaccine expressing the full-length S protein likely facilitated optimal antigen presentation, enhancing the production of neutralizing antibodies at an accelerated rate. Additionally, the lactis vector is known for its ability to effectively deliver antigens and stimulate mucosal immunity, possibly further contributing to the rapid onset of immune responses. In contrast to vaccination schedules in previous studies [48,61,62], our study shows the potential benefits of more intensive immunization protocols in high-risk scenarios like PEDV outbreaks. By increasing the frequency of vaccine doses, we may be able to shorten the time required to generate protective immunity, potentially saving lives in situations where a quick immune response is critical.

*L. lactis* is a promising mucosal delivery vector for vaccines due to its safety profile and ability to express recombinant antigens [63]. However, its practical application is hindered by poor intestinal colonization, which reduces antigen retention and consequently lowers vaccine efficacy [23]. To overcome this limitation, our study encapsulated the recombinant *L. lactis* NZ3900 strain, expressing the full-length S protein of PEDV, in an alginate–chitosan matrix. The encapsulated vaccine demonstrated a greater ability to colonize the intestine (Figure S2), as determined by PCR analysis using primers listed in Table S1, which may have contributed to the significantly enhanced immune response—evidenced by higher levels of PEDV-specific antibodies in mice immunized with the encapsulated vaccine compared to those receiving the non-encapsulated formulation (Figure 3). The alginate–chitosan matrix provides several advantages that address the challenges of *L. lactis*. First, it protects the live bacterial vector and its expressed antigen from the harsh conditions of the gastrointestinal tract, such as acidic pH and digestive enzymes, ensuring a higher survival rate of the bacterial cells as they move from the stomach to the intestine. This protection allows the bacteria to effectively initiate mucosal immune responses in the intestine. Second, the encapsulation enhances the retention time of *L. lactis* within the intestinal environment by improving its adherence to the mucosal lining [64,65]. This prolonged retention facilitates better antigen presentation, stimulating a stronger immune response. Additionally, chitosan’s ability to transiently open epithelial tight junctions promotes antigen translocation to the underlying immune cells, thereby enhancing immune activation [66,67,68,69]. Our results show that animals immunized with the encapsulated recombinant *L. lactis* produced significantly higher levels of PEDV-specific antibodies compared to those vaccinated with the non-encapsulated strain. This enhancement is likely due to the improved stability and delivery efficiency provided by the encapsulation [70,71]. By preserving the viability and bioavailability of *L. lactis* cells, the encapsulation ensures a sustained release of the S protein antigen, crucial for activating both mucosal and systemic immune responses. The rapid progression and high mortality associated with PEDV infections in newborn piglets highlight the urgent need for a fast and potent immune response. The encapsulated vaccine addresses this need by optimizing the delivery and efficacy of *L.* lactis-based vaccines. By inducing higher levels of PEDV-specific antibodies, this strategy enhances both mucosal and systemic immunity, providing better protection against PEDV in a shorter time frame. Therefore, our findings emphasize the potential of alginate–chitosan encapsulation in overcoming the limitations of *L. lactis* as a vaccine delivery vector. By improving the stability, intestinal retention, and antigen delivery, this encapsulated recombinant *L. lactis* vaccine offers significant promise for protecting against PEDV, particularly in high-risk populations like newborn piglets. Moreover, this encapsulation approach could be a valuable enhancement for live bacterial vaccines targeting other enteric pathogens. While our results indicate a promising immune response, the actual protective efficacy of the vaccine prototype against PEDV infection remains to be fully established. Further studies involving challenge experiments with virulent PEDV strains in target animal models, such as piglets, are necessary to validate its effectiveness in real-world scenarios. These experiments would provide more direct evidence of the vaccine’s protective capacity and its potential applicability in the field.

## 5. Conclusions

In conclusion, this study investigates an oral vaccination strategy utilizing *L. lactis* NZ3900 to deliver S epitopes of the PEDV spike protein, with the goal of developing an effective, anti-PEDV oral vaccine. The encapsulated *L. lactis* NZ3900/pNZ8149-S vaccine demonstrated the ability to effectively induce mucosal, humoral, and cellular immune responses against PEDV, underlying its potential as a promising vaccine candidate.

## Figures and Tables

**Table 1:** Lymphocyte proliferation index.

Group	Stimulation Index (SI)	
	S	ConA
Encapsulated *L. lactis* NZ3900/pNZ8149-S	2.40 ± 0.22 c	2.79 ± 0.19 c
*L. lactis* NZ3900/pNZ8149-S	1.92 ± 0.23 b	2.46 ± 0.31 b
*L. lactis* NZ3900/pNZ8149	1.12 ± 0.14 a	1.08 ± 0.12 a
PBS	1.06 ± 0.09 a	0.97 ± 0.13 a

Note: different lowercase letters represent differences among groups (*p* < 0.05) between the assays for the same stimulation. Data are presented as the mean ± SD (*n* = 6).

## Data Availability

Data are available from the authors upon request.

## Supplementary Materials

The following are available online at https://www.mdpi.com/article/10.3390/vaccines13040421/s1, Table S1: Specific primers used in this study, Figure S1: The expression of S protein in Various Generations of recombinant *L. lactis* NZ3900/pNZ8149-S, Figure S2: Evaluation of *L. lactis* NZ3900/pNZ8149-S colonization in mouse gut after oral administration. Refs. [72,73] are cited in Supplementary Materials.
