# Supplementary material for: Oral Delivery of Lactococcus lactis Expressing Full-Length S Protein via Alginate–Chitosan Capsules Induces Immune Protection Against PEDV Infection in Mice
Source: Vaccines (Basel). 2025 Apr 17;13(4):421. doi: 10.3390/vaccines13040421 (PMC12030989; doi:10.3390/vaccines13040421)
Supplement: Supplementary file 1 [file vaccines-13-00421-s001.zip › vaccines-3559103-supplementary.pdf]

Supplementary Materials

Method

Fecal DNA was extracted using the TIANamp Stool DNA Kit (TIANGEN, Beijing, China). Quantitative PCR (qPCR) was performed using target-specific primers for *Lactococcus* spp. as well as universal primers for the *Bacteria* 16S rRNA gene, as listed in Table S1. The qPCR reactions were run on a LightCycler 96 system (Roche, Basel, Switzerland)) under the following conditions: initial denaturation at 95 °C for 15 minutes, followed by 40 amplification cycles consisting of denaturation at 95 °C for 10 seconds, annealing at 60 °C for 20 seconds, and extension at 72 °C for 30 seconds. Each reaction was performed in a final volume of 20 μl in technical duplicates. Relative copy numbers were calculated using the 2^−ΔΔCT method.

Table S1. Specific primers used in this study.

Target group	Primers	Sequence (5′-3′)b	Amplicon Length (bp)	Reference
Bacteria	341F	CCTACGGGAGGCAGCAG		[72]
(rRNA 16S)	518R	ATTACCGCGGCTGCTGG		
Lactococcus sp.	Forward	GAGGCAGCAGTAGGGAATCTTC	133	[73]
	Reverse	CTTGATGAGCTTTCCACTCTCA		

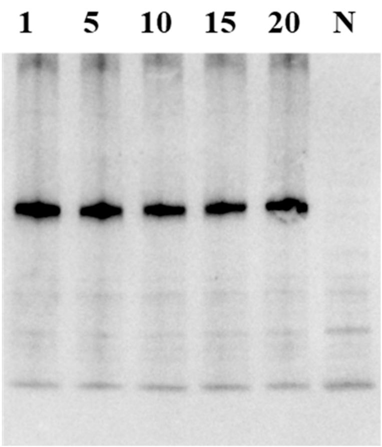

Figure S1. The expression of S protein in Various Generations of recombinant *L. lactis* NZ3900/pNZ8149-S. Lanes 1-20: The expression of S protein from different generations of recombinant *L. lactis* NZ3900/ pNZ8149-S with nisin induction; N: NZ3900 with the plasmid pNZ8149 with nisin induction (negative control).

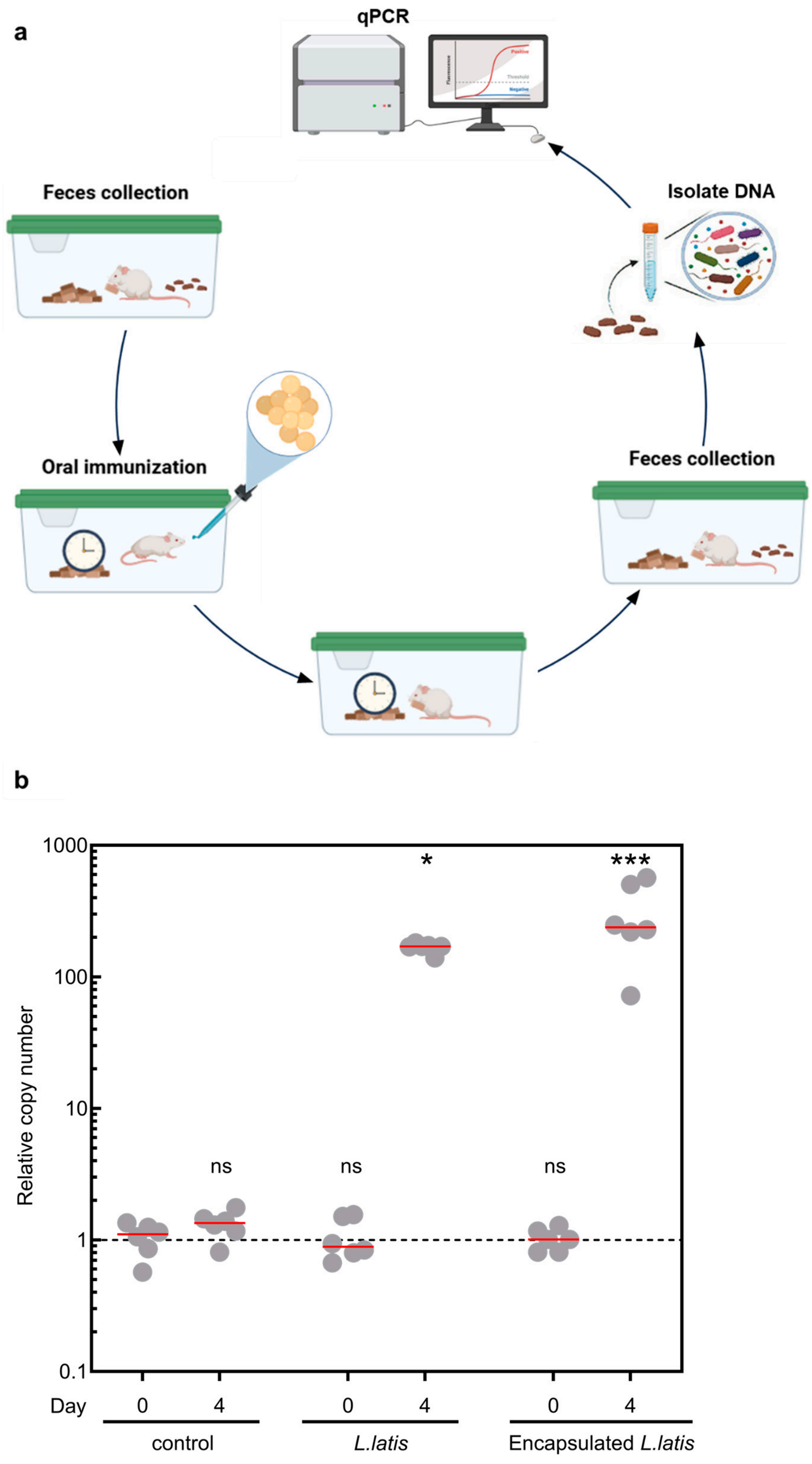

Figure S2. Evaluation of L. lactis NZ3900/pNZ8149-S colonization in mouse gut after oral administration. (a) Outline of the oral *L. lactis* colonization experiment with different treatment (no bacteria, *L. lactis* NZ3900/pNZ8149-S or encapsulated *L. lactis* NZ3900/pNZ8149-S). Four days after the first oral immunization, mice feces were collected, and the *L. lactis* NZ3900/pNZ8149-S load in the gut was quantified. (b) The number of *L. lactis* (number of bacterial genome equivalents) per mouse was determined by qPCR using *L. lactis*-specific 16S rDNA gene primers. Red bars represent the median.
